# Supplementary material for: Clinical and Molecular Features of KRAS-Mutated Lung Cancer Patients Treated with Immune Checkpoint Inhibitors
Source: Cancers (Basel). 2022 Oct 8;14(19):4933. doi: 10.3390/cancers14194933 (PMC9562655; doi:10.3390/cancers14194933)
Supplement: Supplementary file 1 [file cancers-14-04933-s001.zip › cancers-1896306-supplementary.pdf]

List of keywords and abbreviations.

Table S1. Overall Survival and Associations with KRAS Co-mutations.

CI	Confidence interval
CDKN2A/B	Cyclin-dependent kinase inhibitor 2A/B
EGFR	Epidermal growth factor receptor
FANCA	Fanconi anemia complementation group A
GAs	Genomic alterations
HR	Hazard ratio
ICIs	Immune checkpoint inhibitors
IHC	Immunohistochemistry
IRB	Institutional review board
NGS	Next-generation sequencing
NSCC-NOS	Non-small cell carcinoma, not otherwise specified
NSCLC	Non-small cell lung cancer
OS	Overall survival
PD	Progressive disease
PD-1	Program death −1
PD-L1	Programmed death-ligand 1
PR	Partial response
OR	Odds ratio
TMB	Tumor mutation burden
VS	Versus
